# Dropping out of voluntary community-based health insurance in rural Uganda: Evidence from a cross-sectional study in rural south-western Uganda

**DOI:** 10.1371/journal.pone.0253368

**Published:** 2021-07-16

**Authors:** Emmanuel Nshakira-Rukundo, Essa Chanie Mussa, Min Jung Cho

**Affiliations:** 1 Institute for Food and Resource Economics, University of Bonn, Bonn, Germany; 2 Apata Insights, Kampala, Uganda; 3 Department of Agriculture Economics, University of Gondar, Gondar, Ethiopia; 4 Faculty Governance and Global Affairs, Leiden University College, The Hague, Netherlands; University of Georgia, UNITED STATES

## Abstract

**Aim:**

Community Based Health Insurance (CBHI) schemes have become central to health systems financing as avenues of achieving universal health coverage in developing countries. Yet, while emphasis in research and policy has mainly concentrated on enrolment, very little has been apportioned to high rates of dropping out after initial enrolment. The main aim of this study is to understand the factors behind CBHI dropping out through a cross-sectional quantitative research design to gain insights into curtailing the drop out of CBHI in Uganda.

**Methods:**

The survey for the quantitative research component took place between August 2015 and March 2016 covering 464 households with under-5 children in south-western Uganda. To understand the factors associated with dropping out of CBHI, we employ a multivariate logistic regression on a subsample of 251 households who were either currently enrolled or had enrolled at one time and later dropped out.

**Results:**

Overall, we find that 25.1 percent of the households that had ever enrolled in insurance reported dropping out. Household socioeconomic status (wealth) was one of the key factors that associated with dropping out. Larger household sizes and distance from the hospital were significantly associated with dropping out. More socially connected households were less likely to drop out revealing the influence of community social capital in keeping households insured.

**Conclusion:**

The findings have implications for addressing equity and inclusion concerns in community-based health insurance programmes such as one in south-western Uganda. Even when community based informal system aim for inclusion of the poorest, they are not enough and often the poorest of the poor slip into the cracks and remain uninsured or drop out. Moreover, policy interventions toward curtailing high dropout rates should be considered to ensure financial sustainability of CBHI schemes.

## Introduction

Community-based health insurance (CBHI) schemes are a type of private health insurance, often growing from traditional systems of shock coping such as self-help groups and involving substantial community presence in issues such as management and premium setting [1,2]. Their foundations in traditional risk coping systems brings in the characteristic of mutual aid and risk pooling that is often not the case in other types of health insurance such as private and social health insurance. Over the last couple of decades, CBHI schemes have become central to health systems financing in the post Alma Ata declaration era of achieving universal health coverage [1,3,4]. Many developing countries have therefore invested in such schemes to provide pre-payment based health services to their populations. While the number of these schemes have inarguably exponentially increased, enrolment of the target population has remained low hence attracting a lot research in understanding why enrolment [5,6] and willingness to pay for these schemes [7] remain low.

However, not only is enrolment low but dropping out of CBHI after initial enrolment also remains high. For instance, in Ghana, recent evidence shows increasing dropout, higher than 50 percent [8], a sharp increase from earlier recording of 34 percent [9]. In Burkina Faso, early evaluations indicated dropout rates between 30 and 45 percent [10] while in Senegal, rates of up to 83 percent were recorded [11]. In Sudan, 40 percent dropped out [12] while in Ethiopia, up to 27 percent did [13]. In India, Panda and colleagues [14] study schemes operating in Bihar and Uttar Pradesh and report re-enrolment rates of less than 20 percent, implying dropout rates of as much as 80 percent. Moreover, another scheme in Ahmedabad, India recorded 63 percent drop outs [15]. In most cases, the proportion of dropouts is higher than new enrolment hence low net participation. Despite these, jaw-dropping statistics, in comparison to research on enrolment, studies investigating dropping out of CBHI schemes are still very few. From these studies, the major drivers of dropping out are high premiums [9,12,13], restricted benefits packages [9], perceived poor quality of services [9–11] and poor understanding of insurance concepts [12,13].

Suffice to mention that there is a growing interest in health insurance in Uganda, especially the recent growth of CBHI schemes across the country [16–19]. In this particular study, we found that 44 percent of the households were enrolled in CBHI [20]. Previous studies however, focussed on low enrolment [21,22] and no study so far has investigated dropping out decisions. Our main contribution with this study is therefore to extend research in understanding the factors for high insurance dropping out. This study comes at a critical juncture especially for the government of Uganda as it plans to roll out the national health insurance scheme. The scheme is expected to incorporate existing CBHI schemes into the national risk pool in a progressively manner [23] so devising strategies of limiting dropping out will be crucial for the scheme success. This study informs both academia and policy spaces of challenges of maintaining insurance once individuals or households have enrolled and attaining universal health coverage primarily using CBHI in Uganda and other developing countries.

## Methods

### Study setting: Kisiizi community-based health insurance scheme sample characteristics

The Kisiizi CBHI scheme is the largest scheme in Uganda. Having started in 1996, the scheme currently covers over 42,000 individuals in close to 10,000 households. Insurance premiums are levied according to household size, with decreasing unit cost per additional household member. In 2015 and early 2016 (at the time of data collection), each individual above 6 months of age paid annual premiums ranging from $3 (Uganda shillings 10,000) per person for households of 7–11 individuals to $8 (Uganda shillings 30,000) per person for households of 1–2 individuals. Accordingly, these premiums were equivalent to about 2 percent of annual income in south-western Uganda, based on the 2016 assessments [24].

A special feature of the Kisiizi CBHI scheme is its origins and continuous linkages with informal traditional social support systems in form of burial groups. Burial groups are essentially kin-associated arrangements of households where members support each other in case of bereavement of a household member. These groups are known to have been present for many decades [25,26]. Virtually, every household belongs to one and non-membership often attracted social punishments and sanctions [25]. Enrolment into the Kisiizi CBHI scheme is dependent on current membership in a traditional burial group of at least 30 households. For larger burial groups for which 60 percent is at least 30 households, at least 60 percent of the group is necessary for group members to enrol. These conditions are put in place to reduce moral hazard and adverse selection [27]. The scheme covers basic primary care, maternity care, surgeries, and outpatient and inpatient services, up to a maximum coverage of about $500 per hospitalisation episode. Excluded services are outpatient services for chronic illnesses and substance abuse related illnesses and injuries. Readers are referred to recent studies [28–31] for a detailed overview of this scheme.

### Sampling and data collection

Overall, Kisiizi CBHI scheme operates in four districts (Kabale, Kanungu, Rukungiri and Ntungamo) in south-western Uganda. However, due to the abolition of user fees in public health facilities in Uganda [32,33], health insurance of any kind is only provided in private health facilities. However, large section of the rural population does not have access to public/ government health facilities and therefore depend on private health facilities. Of the 163 district hospitals in the country in 2018, only 41 percent were public/ government hospitals [34]. Our sample was from three sub-districts (referred to as sub-counties in Uganda) in Rukungiri and Kabale, services by one private hospital, Kisiizi hospital.

Our sample was drawn from 14 randomly selected villages in Nyarushanje and Nyakishenyi sub-counties in Rukungiri district and Kashambya sub-county in Kabale district. In each village, with the assistance of village leaders, a household list consisting of households with at least one child between 6 and 59 months was drawn and all households were eligible for survey administration. The questionnaire included a household demographics module, a socioeconomic characteristics module, food and nutrition module, household assets, a health module and a CBHI module. The survey mainly targeted households with an under-five child as households with these characteristics were the focal group of the overall study. Due to possibility of recall errors, we collected child health information for only the youngest child in the households whenever there were more than one under-five children. The survey was administered by four trained female enumerators since it covered other sensitive topics on women’s health. The questionnaire was developed by the researchers and pretested in one village on a sample of 30 respondents. Altogether, the survey was administered to 464 households between August 2015 and March 2016. In this paper, we use a sub-sample of 251 households that enrolled in insurance at the time of the survey or had enrolled at one time and dropped out. The survey was administered using a Computer Assisted Personal Interviewing (CAPI) platform that was able to provide real time data with fewer errors.

### Data analysis

To understand the factors associated with dropping out of CBHI, we estimate a simple logistic regression model built in a hierarchical order. The first model considers only household socioeconomic variables. The second model adds social connectivity and social capital variables and the third model includes community variables that assist accounting for community heterogeneity. The takes the form below.


$$Y_{it}=\beta_{0}+H_{it}\beta_{1}X_{it}\beta_{2}+\Gamma_{it}\beta_{3}+\varepsilon_{i}$$

Where ***Y_i_*** is a binary outcome variable taking the value 1 if a household *i* in village *t* ever dropped out of CBHI and 0 if the household was always enrolled. **H_*it*_** is the vector of household socioeconomic and demographic controls, **X_*it*_** is a vector of social network and perception indicators and **Γ_*it*_** is a vector of community characteristics such as distance to hospital and altitude of the village. In order to reduce collinearity and improve model fitting, we follow Iacobucci et al [35] approach to mean centering our predictors namely, child’s age in years, household size, household diet diversity score, number of antenatal care visits, number of burial groups in the village, distance to hospital and village altitude. To check and account for possible multi-collinearity between the controls, we compute the variance inflation factor and show the test statistic. Going by the most common rule of thumb of 10 [36], we ascertain that the inclusion of additional blocks of variables does not induce multi-collinearity. A list of all variables and the corresponding vectors in which they belong is in supplementary materials (S2 File variable description).

The key outcome is dropping out of CBHI. We assess dropping out in a less restrictive way but also show results (in S3 File) of a more restrictive assessment. In the less restrictive assessment, which is the primary measure, we asked respondents if they had ever dropped out CBHI since its inception. This helps us to capture pre 2010 dropping out prevalence. We then asked respondents of their enrolment status in 2010. Dropping out is then defined as one-zero dummy where 1 is having ever dropped out pre-2010 and also having dropped out between 2010 and 2015 and zero is current enrolment without ever dropping out. This is a less restrictive assessment. An alternative, more restrictive assessment does not consider the pre-2010 household enrolment behaviour and only compares the two periods plus new enrolment.

## Results

### Descriptive results

**Fig 1:**
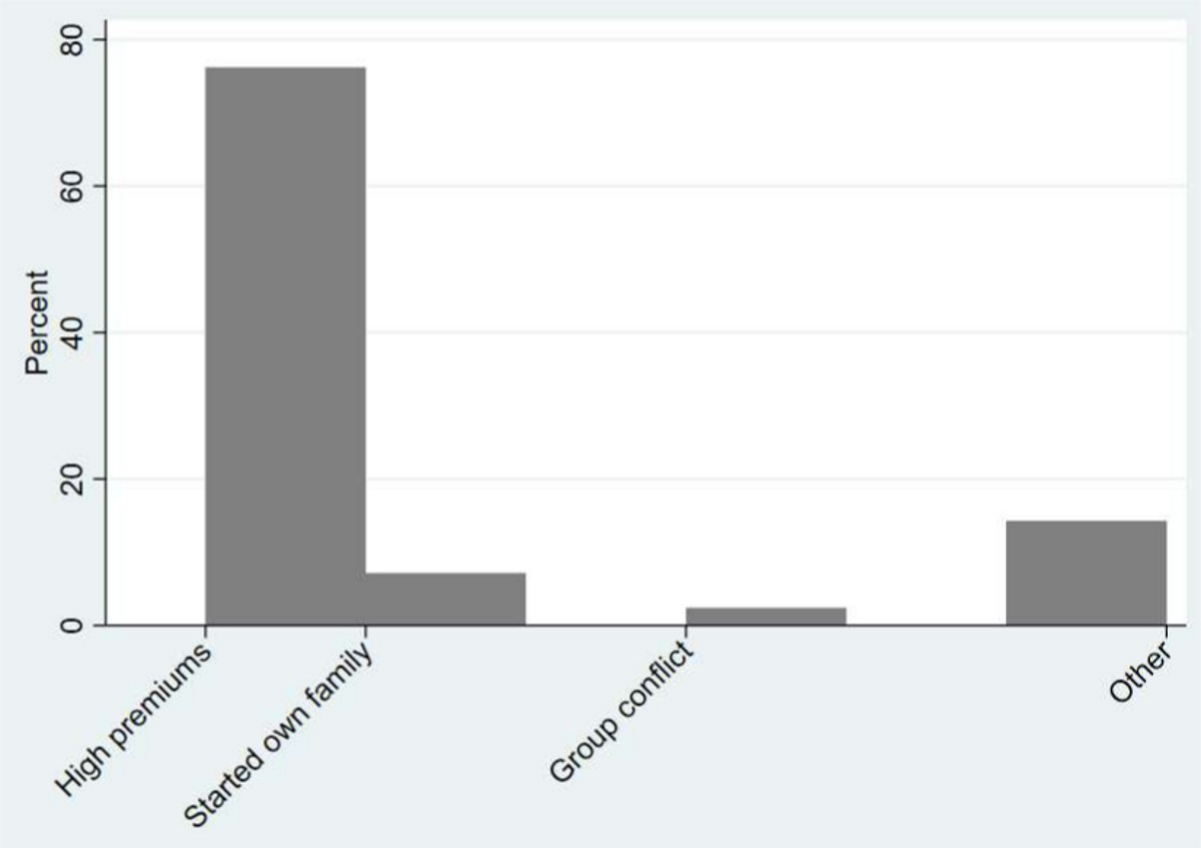
Reasons for dropping out of CBHI.

Overall, in a sample of 251 households, 25.1 percent had ever dropped out of CBHI. Fig 1 below shows the reasons for dropping out.

About 76 percent had stated high premiums as the main reason for dropping out. When an individual started their own household, they would be removed from their parents’ household schedule and hence must enrol afresh. About 7 percent of the households stated starting their own household as a reason for dropping out. This reason is also related to affordability of premiums in that younger people generally face challenges of remaining insured when they are no longer covered/paid for by their parents. We show other summary statistics of the all households in the analysis in Table 1 below. To show a more refined comparison, we show mean comparison t-tests that compare households that dropped out and households that were insured and never dropped out (column 3).

**Table 1:** Summary statistics.

	Never dropped		Dropped out		Difference	
	mean	sd	mean	sd	Mean diff	t-stat
Mother’s age	30.129	7.189	31.953	6.717	-1.824*	(-1.77)
Father secondary education	0.165	0.372	0.222	0.419	-0.057	(-1.02)
Mother secondary education	0.144	0.352	0.175	0.383	-0.031	(-0.59)
Catholic	0.644	0.480	0.635	0.485	0.009	(0.12)
Married	0.936	0. 245	0.921	0.272	0.016	(0.42)
Household size	5.234	1.820	5.984	2.044	-0.750***	(-2.74)
Father casual employment	0.410	0.493	0.397	0.493	0.013	(0.18)
Household diet diversity score	4.207	1.310	4.095	1.027	0.112	(0.62)
Food adequacy	0.559	0.498	0.460	0.502	0.098	(1.35)
Wealth index						
Quintile 1 (Poorest)	0.144	0.352	0.254	0.439	-0.110**	(-2.02)
Quintile 2	0.181	0.386	0.333	0.475	-0.152**	(-2.55)
Quintile 3	0.245	0.431	0.127	0.336	0.118**	(1.97)
Quintile 4	0.266	0.443	0.175	0.383	0.091	(1.46)
Quintile 5 (Richest)	0.165	0.372	0.111	0.317	0.054	(1.03)
Satisfaction index	0.166	2.768	-0.013	3.262	0.179	(0.42)
Burial group size	59.553	22.114	67.905	22.630	-8.352**	(-2.58)
Neighbour in CBHI	0.910	0.288	0.746	0.439	0.164***	(3.39)
Number of other voluntary groups	9.697	2.464	7.952	3.139	1.744***	(4.52)
Information access	2.411	0.829	1.873	0.871	0.538***	(4.40)
Waiting time	0.660	0.475	0.556	0.501	0.104	(1.48)
Perceptions about CBHI						
Scheme management	115.122	124.903	92.302	104.903	22.82	(1.30)
Social influence	0.407	1.497	0.305	1.707	0.102	(0.45)
Health beliefs	0.336	1.349	0.057	1.440	0.279	(1.40)
No of burial groups in village	0.148	1.881	-0.014	2.296	0.162	(0.56)
Village has a traditional birth attendant	0.287	0.454	0.476	0.503	-0.189***	(-2.78)
Village has a health centre	0.319	0.467	0.333	0.475	-0.014	(-0.21)
Village has a school	0.585	0.494	0.524	0.503	0.061	(0.85)
Distance to hospital (kms)	9.493	2.934	10.965	2.757	-1.472***	(-3.50)
Village altitude (metres)	1782.159	88.499	1723.065	96.576	59.09***	(4.48)
*N*	188		63		251	

t-test significance of the mean difference

* p<0.10

** p<0.05

*** p<0.01.

We observe that in majority of the variables included in our analysis, households dropping out CBHI were significantly different from those that remained enrolled. Households that dropped out of CBHI were more likely to have older mothers, older by about 1.8 years compared to that never dropped out. Households that dropped out had about 0.75 persons more that those that never dropped out.

Households remaining enrolled were more likely to have a neighbour in insurance than those that dropped out. A similar pattern is also observed regarding membership in other voluntary groups in the village. Households that never dropped out CBHI belonged to remained to 0.54 more groups than those that enrolled and dropped out. However, regarding the size of burial groups, there was a difference of about 8.4 households between those that dropped out and those that remained enrolled. The implication here seems to be that while non-CBHI households belong in fewer social support groups, they tend to be part of larger burial groups that provide extended social support that could substitute the necessity for formally enrolling in CBHI. On the other hand, households that remain in CBHI seems to belong to relatively smaller burial groups for which it might be easier to maintain consensus about the CBHI enrolment agenda while maintaining core burial groups functions of informal funeral insurance. Using the principal components analysis, a method used to reduce many correlated variables into a single index variable [37], we developed several indices to capture key dimensions of households responses. First, we develop a wealth index that ranks households from the richest to the poorest based on household’s asset holding. The index was then divided into 5 equivalent quintiles. Among the poorest quintile, there were 11 percent more households that dropped out than those that never did. Among the second poorest quintile, the difference between those that dropped out and those that did not was 15 percent. However, in the average quintile, there were fewer households who dropped out than those that remained insured. A larger proportion of households that dropped out were more likely to be in the bottom two quintiles (poorest or poor) while there were no significant differences in households in the richer categories.

We then use the same principal components method to develop three perception indices that measure respondents’ perceptions regarding (1) management of the CBHI scheme, (2) social influence by family, friends and neighbours to enrol in CBHI, and (3) health beliefs. This method has been used in other health insurance perception studies [38] and the questions we use are similar to those employed by Jehu-Appiah and colleagues in Ghana [38]. We observe that households that never dropped out reported generally more positive perceptions regarding scheme management and social influence when compared to those that dropped out or those that never enrolled. We do not observe any significant differences between any of the comparisons with households that dropped out.

Finally, we highlight on the characteristics of villages in which these groups of households are. We observe three significant differences between dropping out and staying insured by village of residence. First, households that stayed insured had close to two more burial groups per village than those that dropped out. It is important to remember that enrolling in insurance is a function of participation and membership in burial groups so the more the burial groups in the village the higher the likelihood of enrolling and remaining enrolled. Forty-seven percent of households that dropped out of were in villages with a traditional birth attendant. However, only 29 percent of households that did not drop out had a traditional birth attendant in their villages, revealing a mean difference of 18.9 percent. Additionally, households that dropped out lived in villages further from the hospital by 1.5 kilometres compared to households that did not drop out.

### Regression results

In order to understand the factors that influence dropping out of CBHI, we conduct hierarchical logistic regressions. In Table 2, we show results of adjusted odd ratios that predict the likelihood of dropping out of CBHI. We present the comparison between households that dropped out after enrolment and those that remained enrolled and never reported ever dropping out of CBHI at the time of data collection.

**Table 2:** Nested logistic regression results of correlates of dropping out of CBHI.

	(Model 1)		(Model 2)		(Model 3)	
VARIABLES	Odds Ratio	95% CI	Odds Ratio	95% CI	Odds Ratio	95% CI
Mother’s age	1.016	0.972–1.061	1.061**	1.007–1.119	1.055*	0.994–1.120
Father secondary education	2.864**	1.169–7.018	3.479***	1.355–8.933	2.756*	1.000–7.596
Mother secondary education	1.902	0.757–4.779	1.752	0.587–5.227	1.370	0.459–4.090
Catholic	0.915	0.494–1.695	1.138	0.595–2.176	1.651	0.747–3.648
Married	0.735	0.216–2.500	0.588	0.158–2.188	0.555	0.151–2.043
Household size	1.280***	1.071–1.530	1.117	0.918–1.359	1.131	0.891–1.435
Father casual employment	0.734	0.353–1.524	0.770	0.344–1.721	0.856	0.347–2.110
HDDS	1.068	0.799–1.429	1.081	0.786–1.486	1.021	0.671–1.553
Food adequacy	0.823	0.397–1.708	0.939	0.436–2.021	0.941	0.405–2.184
Wealth index (base Q5 -richest)						
Quintile 1 (poorest)	6.912***	2.053–23.274	6.747**	1.544–29.477	9.087**	1.602–51.538
Quintile 2	7.213***	2.250–23.126	9.426***	2.357–37.690	12.813***	2.666–61.570
Quintile 3	1.380	0.441–4.318	1.594	0.418–6.080	2.675	0.503–14.213
Quintile 4	1.644	0.586–4.610	2.196	0.665–7.252	3.284	0.622–17.337
Information access	0.863	0.432–1.724	0.911	0.439–1.892	0.991	0.451–2.180
Waiting time	0.998	0.995–1.001	0.998	0.995–1.002	0.997	0.994–1.001
Satisfaction index			0.971	0.710–1.329	0.947	0.680–1.318
Burial group size			1.019***	1.005–1.032	1.029**	1.007–1.051
Neighbour in CBHI			0.376**	0.168–0.839	1.095	0.413–2.903
Number of other voluntary groups			0.391***	0.227–0.671	0.449***	0.254–0.794
Perceptions about CBHI						
Scheme management			1.030	0.800–1.326	1.011	0.763–1.342
Social influence			0.930	0.742–1.166	0.872	0.697–1.091
Health beliefs			0.937	0.608–1.443	0.924	0.578–1.475
No of burial groups in village					0.741***	0.598–0.917
Village has a traditional birth attendant					1.591	0.735–3.441
Village has a health centre					0.839	0.172–4.086
Village has a school					3.002	0.501–17.998
Distance to hospital (kms)					1.211*	0.969–1.515
Village altitude (metres)					1.001	0.994–1.007
Constant	0.050***	0.009–0.279	1.507	0.158–14.412	0.210	0.011–4.134
Observations	251		251		251	
R-squared	0.125		0.233		0.307	
Mean VIF	3.29		4.14		4.29	

Robust standard errors in parentheses

*** p<0.01

** p<0.05

* p<0.1.

Model 1 shows household socioeconomic and demographic correlates of dropping out. We observe that father secondary education (OR 2.9; 95%CI 1.169–7.018), being in a larger household (OR 1.3; 95%CI 1.071–1.530) and being poor in either the poorest quintile (OR 6.9; 95%CI 2.053–23.274) or the second poorest (OR 7.2; 95% CI 2.250–23.126) were correlated with dropping out of CBHI. In Model 2, once we add social connectivity and perception variables, we observe that the odds of dropping out are almost 3.5 times higher in households in which the father was educated up to secondary school level (OR 3.479; 95% CI 1.355–8.933). The odds of dropping out are also 6.7 times for the poorest households (OR 6.747; 95% CI 1.544–29.477) and over 9 times higher for poor households (OR 9.426; 95% CI 2.357–37.690). We also observe that and additional year of mothers age was associated with 6% higher likelihood of dropping out (OR 1.061; 95% CI 1.007–1.119) and an additional household in a burial group increased the odds of dropping out by almost 2 percent (OR 1.019; 95% CI 1.005–1.032). Two social connectivity variables reduce the odds of dropping out. Having a neighbour in CBHI reduced the odds of dropping out by over 66 percent (OR 0.376; 95% CI 0.168–0.839) while belonging to one additional voluntary self-help group also reduced the odds of dropping out by just under 70 percent (OR 0.391; 95% CI 0.227–0.671).

Model 3 adds village variables to account for across villages’ heterogeneity. Controlling for village level variation, we observe the consistent correlation of mother age (OR 1.055; 95% CI 0.994–1.120) and father education (OR 2.756; 95% CI 1.000–7.596) in dropping out. Similarly, the odds of poorer households dropping out were higher, over 9 times for the poorest (OR 9.087; 1.602–51.538) and close to 13 times for poor households (12.813; 95%CI 2.666–61.570). An additional household in a burial groups increased odds of dropping out by 2.9 percent (OR 1.029; 95% CI 1.007–1.051) and one additional kilometre from the hospital increased the odds of dropping out by 21 percent (OR 1.211; 95% CI 0.969–1.515). Belonging to one additional voluntary self-help group reduced the odds of dropping out by 55 percent (OR 0.449; 95% CI 0.254–0.794) while an additional burial group in the village reduced the odds of dropping out by about 26 percent (OR 0.741; 95% CI 0.598–0.917).

## Discussion

We find a dropout rate of about 25 percent. In general this prevalence of dropping out was lower than other voluntary health insurance programmes such as Ghana [8,9], Burkina Faso [10] and Ethiopia [13]. However, these are not convincingly low prevalence rates either. We find that poorer, socioeconomically worse-off households are more likely to drop out of health insurance compared to richer households. This result is not surprising and collaborates other studies that found that higher premium rates were among the leading causes of dropping out [9,12,13]. Though our results disagree with Mladovsky [11] who did not observe any association of household financial capacity with dropping out of CBHI in Senegal, close to 80 percent of our sample who dropped out mentioned inability to afford premiums as the main reason. A study from Ghana [8] also confirms that while the poor are always the quickest to enrol, they are also the fastest to drop out implying that the need for protection is often inhibited with the financial capacity to afford premiums. Indeed, financial capacity is highly in line with household size such that, as we observe, larger households are more likely to drop out compared to smaller size households.

Our results do not reveal a strong correlation with dissatisfaction with the quality of health services or the perception of health insurance in general though these have been observed elsewhere [39–43]. Previous qualitative studies in Uganda revealed low understanding of health insurance from community members [21,22]. This is therefore an issue that requires more research. We cannot conclude, with our small sample that client perceptions do not affect dropout behaviour.

While household factors are central, community social capital and network factors also play an important role in keeping households enrolled. Our results here are in two dimensions. We observed, on the one hand, that belonging to more voluntary self-help groups and having a neighbour participating in CBHI reduced the likelihood of dropping out. When households belong to more than one social support groups, they enlarge their social support circle. For instance, members are able to lend and borrow in voluntary savings and loans associations, hence mitigating possible dropout. In the same manner, neighbours in CBHI are more likely to influence each other’s’ behaviour, possibly with positive social pressure such that households who remain insured instigate their neighbours to remain insured as well.

However, the effect of burial groups is multi-dimensional. First, we found that households in large burial groups were more likely to drop out but an additional burial group in the village reduced dropping likelihood. These two results should be read in view of the role burial groups generally play in people’s livelihoods. Burial groups have existed in south-western Uganda for centuries and membership is often non-negotiable, attracting social sanctions for defaulting members [25]. Beyond their key role of group-based informal funeral insurance [44,45], burial groups play a broader social support role in developing countries [46,47]. In south-western Uganda, these groups are used as key organising platforms for health insurance [27]. The finding that an additional burial group reduces the odds of dropping out of insurance therefore implies that burial groups facilitate enrolment as well as reducing dropping out.

However, we find that if a burial group becomes bigger, members might become dis-incentivised to stay in CBHI. It is important to understand that the formative purposes of burial groups are informal risk pooling [48], therefore larger burial groups with a larger risk-pooling network are likely preferred by many households. Moreover, in larger group sizes, free riding is likely to happen [49] and group social capital is likely to decline [17] hence increasing the risk of dropping out. In addition, for insurance organisation purposes, group leaders are more likely to prefer smaller groups in which control and management are efficient. Larger groups are therefore more likely to separate into more groups to keep management efficient. These two results therefore do not contradict but rather bring to attention the key role of smaller, better-managed burial groups in the course of enlarging insurance membership and keeping households enrolled.

One intriguing issue is the role of fathers’ education for which we found that households with more educated (in our case having at least some secondary education) fathers were more likely to drop out of CBHI. We are not entirely sure why this effect is present and strong since other studies show that more education households are more likely to renew and less likely to drop out [9,10,13]. Our postulation is that education is correlated with migration to urban areas such that households whose main income earners migrate to urban areas for work reasons are less likely to remain enrolled. Two mechanisms might be at play. First, such households might have more incomes and therefore prefer fast and efficient out of pocket care as opposed to a bureaucratic insurance-based care. Secondly, since health insurance is strongly linked to community links, households whose heads migrate might lose some of these important community links hence increasing their odds of dropping out. Unfortunately, we do not have enough data to test these postulations.

## Conclusion

This study makes important contributions to a handful of studies on dropping out of voluntary CBHI in developing countries. This important area deserves more research in order to ensure the sustainability of emerging health financing policies in these countries. If these schemes are not sustainably keeping individuals insured, they might not be dependable for the health system. Our findings suggest that factors influencing CBHI drop out are mainly of financial nature but other factors especially social capital and connectivity play a role. The findings of this study have implications for addressing health service quality of care, especially incorporating feedback from clients into the manner in which health care workers interact with clients. While CBHI-linked burial groups provide mechanisms for support of poorer households, these are not enough and the poorest still fall into the cracks and fail to enrol or drop out due to financial inability.

That wealthier people are less likely to drop out and that households face significant hurdles in meeting premium demands suggests that even when CBHI has some mechanisms of protecting the poor, these are not enough to prevent exclusion of the needy and the poor. Even regarding enrolment, evidence from this particular scheme shows that poor households are less likely to enrol [20]. While there are per-head discounts on household premiums, these are not enough for very poor households. The sustainability of CBHI and protection of the poorest households therefore needs to consider other sources of subsidising the poorest. We believe this could be an opportunity the government of Uganda might consider taking, in the process of incorporating CBHI into the wider planned National Health Insurance Scheme. On the onset of a national health insurance programme, the government of Uganda and other insurance support organisations might consider providing means-tested subsidies to the poorest, as has been implemented in Rwanda [50].

## Supporting information

S1 File Survey questionnaires. (PDF)

S2 File Variable description. (DOCX)

S3 File Regression results with a relaxed sample. (DOCX)
