# Supplementary material for: Dropping out of voluntary community-based health insurance in rural Uganda: Evidence from a cross-sectional study in rural south-western Uganda
Source: PLoS One. 2021 Jul 16;16(7):e0253368. doi: 10.1371/journal.pone.0253368 (PMC8284644; doi:10.1371/journal.pone.0253368)
Supplement: S1 File — (PDF) [file pone.0253368.s001.pdf]

Questionnaire template

Field	Question	Answer
Introduction	Good morning/afternoon. I am [NAME OF ENUMERATOR]. I am part of a research team lead by Mr. Emmanuel Rukundo from Kisiizi hospital in partnership with the Center for Development Research (ZEF), Bonn. You are being invited to participate in this research that aims at understanding whether and how health insurance status of parents (mothers) influences the health of children. This is research will have broader lessons on ways to deliver better health in the regions served by this hospital as well as the country at large.<br>Before you take part in this study, we would like to explain to you some guidelines and your rights to participate. I will read to you these guidelines and the discussion with you will only commence when you have given full consent to participate.<br>Our discussion with you will last between 60 to 90 minutes. We value your opinion and there are no wrong answers to the questions we will be asking in the interview. We shall ask you questions about your health, education, agriculture and other relevant issues about your household. There will be no cost to you other than your time and there will be no direct benefits for participation. However, your participation will help the research team and Kisiizi hospital and other healthcare stakeholders to better understand if health insurance improves the health of those who are insured.<br>Your participation in this research is completely voluntary and confidential. You are free to withdraw your consent and stop the interview at any time, or not answer any particular questions. While through the interview we will ask you your names or names of children in this house, all identities will be coded and stored anonymously. All information we obtain from you during this interview will be kept strictly confidential and your answers will never be shared with anyone other than the research team.<br>Reports which will be published from this research will be shared with Kisiizi hospital, Mengo Hospital Research Ethics Committee and Uganda National Council of Science and Technology (UNCST). In all these, your confidentiality will be guaranteed.<br>If you have any questions regarding your participation in this study, please ask the Principal Investigator at (phone number). Your participation is highly appreciated.<br><br>Declaration of consent:<br>The researcher read to me orally the consent form and explained to me what it means, my rights, benefits of participation. I agree to take part in this research. I understand that I am free to discontinue participation at any time if I so choose, and that the investigator will gladly answer any question that arise during the course of the research.<br>MAY I START NOW?<br>• Yes, if permission is given □ then begin the interview.<br><br>• No, if permission is not given □ Discuss this result with the Principal Investigator.<br>Read this letter of consent to the approached prospective participant	
Questions about the location of the household		
consent *(required)*	May I continue?	O 1 Yes<br>O 0 No
hhid	Enter household number<br>*Response constrained to: .>=001 and .<=650*	
district *(required)*	Name of District	1= Rukungiri<br>2= Kabale

Field	Question	Answer
subcounty *(required)*	Name of Sub county	1=Nyarushanje<br>2=Nyakishenyi<br>3= Kashambya
parish *(required)*	Name of Parish	Names of 9 parishes are redacted for privacy
village *(required)*	Name of the village	Names of 14 villages are redacted for privacy
hhGPS *(required)*	Standard GPS location of the household<br>*Make sure the location precision is less than 25 metres*	
We would like to thank you for accepting to participate in this interview. Now I will ask you about this household's kinship		
hhheadname *(required)*	What is the name of the head of this household?	
mothersname	What is the name of the mother being interviewed?	
mothersdob	When were you born?<br>*If not sure of the exact date, record 1st of the month. If not sure of the month, record 1st of January of the Year*	
maritalstatus	What is your current marital status?<br>*if respondents do not understand, read to them the possible answers*	1 Married<br>2 Divorced or Seperated<br>3 Widowed<br>4 Never married
religion	What is your religion	1 Catholic<br>2 Protestant<br>3 Pentecostal<br>4 Muslim<br>5 Seventh Day Adventist<br>6 Other
userepeat *(required)*	Would you like to use a repeating group for the household roster?<br>*A repeating group has a different UI and data structure.*<br>*Question relevant when: ${consent} =1*	1 Yes<br>0 No
sectionnote	We would like to make a list of household members most importantly, those who are between 6 and 59 months (five years and below).	
numfamily *(required)*	How many children of under five years do you have and live with you?<br>*These have to be children whom the person being interviewed is the mother*<br>*Question relevant when: ${consent} =1 and ${userepeat} =1*<br>*Response constrained to: .>=0 and .<=5*	

Field	Question	Answer
Please list all the children who are below five years in this household		
fam_name1 *(required)*	What is the name of the first other child in the household?<br>*Question relevant when: ${consent} =1 and ${userepeat} =1 and ${numfamily} >=1*	
fam_name2 *(required)*	What is the name of the second other child in the household?<br>*Question relevant when: ${consent} =1 and ${userepeat} =1 and ${numfamily} >=2*	
fam_name3 *(required)*	What is the name of the third other child in the household?<br>*Question relevant when: ${consent} =1 and ${userepeat} =1 and ${numfamily} >=3*	
fam_name4 *(required)*	What is the name of the fourth other fchild in the household?<br>*Question relevant when: ${consent} =1 and ${userepeat} =1 and ${numfamily} >=4*	
rosterpart2note	Now I will ask you a few more questions about each of these family members.<br>*Question relevant when: ${consent} =1 and ${userepeat} =1*	
More questions about child 1<br>*Group relevant when: ${consent} =1 and ${userepeat} =1 and ${numfamily} >=1*		
fam_gender1	What is [fam_name1]'s gender?	1 Male<br>0 Female
fam_dob1	What is [fam_name1]'s date of birth?	
fam_dobcert1	Does [fam_name1] have a birth certificate?	1 Yes<br>2 No<br>3 Don't know
Questions about older children and child mortality		
olderchildren	Do you have any other children who are above the age of five years?	1 Yes<br>0 No
olderchildrennumber	If Yes, how many?<br>*Question relevant when: ${consent} =1 and ${olderchildren} =1*	
childdeath	Are there other children in this household who died after delivery, who by this time would be 5 years or below?	1 Yes<br>0 No
childdeathnumber	If Yes, how many<br>*Question relevant when: ${consent} =1 and ${childdeath} =1*	
sectionote	Now we would like to ask you questions regarding your health, nutrition and later about the status and knowledge of insurance	
Questions about household access to food		
hhmeals	How many meals did members of this household eat yesterday?	1 Three Meals<br>2 Two Meals<br>3 One Meal

Field	Question	Answer
avemealspday	On average, how many meals per day did members of this household have over the last 7 days	1 Three Meals<br>2 Two Meals<br>3 One Meal
u5breakfastmeal	For all children less than 5 years in this household, what meal did they have for breakfast yesterday?	1 Milk tea with sugar<br>2 Tea with sugar<br>3 Solid food only<br>4 Solid food with tea<br>5 Tea without sugar but with solid food<br>6 Porridge with sugar<br>7 Porridge without sugar<br>8 Porridge with solid food<br>9 Porridge with milk<br>10 Other<br>11 Nothing
generated_note_name_79	Now we would like to ask you about your daily food intake using yesterday as an example. We are interested in knowing if your child had in the last 24 hours, any of the following foods and drink that I will mention<br>*Here, you seek to know the household diet diversity scores (HDDS) of the household*	
Questions about household diet diversity scores		
beverages	Did your child (ren) drink any of the following beverages yesterday?	1 Fresh Juice<br>2 Soup<br>3 Obushera<br>4 Black tea or coffee<br>5 Plain water<br>6 Other
milkproducts	Did your child (ren) have milk yesterday?	1 Milk (tinned or other)<br>2 Infant baby fomula
meat	Did your child (ren) eat meat and or other meat products yesterday?	1 Goat, beef, lamb or pork<br>2 liver, kidney or heart
poultry	Did your child (ren)eat any poultry products yesterday?	1 Chicken, duck, turkey, goose etc<br>2 Eggs
fish	Did your child (ren) eat fish or fish products yesterday?	1 Yes<br>0 No

Field	Question	Answer
vegetables	Did your child (ren) eat any vegetables yesterday?	1 Dark green vegetables eg amaranths, spinach, cassava leaves, bean leaves, pumpkin leaves<br>2 Orange coloured vegetables (pumpkim, carrots)<br>3 Bio-fortified food (eg sweet potatoes)<br>4 Other vegetables (cabbages, egg plants, tomatoes)
cereals	Did your child (ren) eat cereals or grains yesterday?	1 Rice, posho, porridge, bread, chapatti, pasta/macaroni, noodles or other foods made from maize, millet, sorghum or other grains such as mandazi, doughnut, pancakes etc<br>2 Other foods made from grains such as weetabix, cornflakes etc
legumes	Did your child (ren) eat any legumes such as beans, peas or groundnuts yesterday?	1 Yes<br>0 No
plantian	Did your child (ren) have any plantian yesterday (eg matooke, kabalagala, gonja, bogoya)	1 Yes<br>0 No
rootsandtubers	Did your child (ren) eat any roots or tubers yesterday such as Cassava, yams, white sweet potatoes or Irish potatoes?	1 Yes<br>0 No
oilsandfats	Did your child (ren) eat any oils and fats such as cooking oil, margerine, blue band, butter etc?	1 Yes<br>0 No
sugar	Did your child (ren) take any sugar and other sugar products such as sweets, chocolate, cakes, cookies etc yesterday?	1 Yes<br>0 No
foodsufficient	Do you think the food that you had over the last week was sufficient and adquate for this household?	1 Surplus<br>2 Always adequate<br>3 Sometimes not adequate<br>4 Not enough<br>5 Very little
generated_note_name_96	Now we would like to ask you about use of mosquito nets in this household	
Now we would like to ask you about use of mosquito nets and malaria control in this household		

Field	Question	Answer
spray	At any time in the past 12 months, has anyone come into your dwelling to spray the interior walls against mosquitoes?	1 Yes<br>2 No<br>3 Don't know
mosquitonet	Does your household have any mosquito nets that are used while sleeping?	1 Yes<br>0 No
netnumber	How many mosquito nets does the household have?<br>*Question relevant when: ${consent} =1 and ${mosquitonet} =1*	
netuse	Who in this household slept under a mosquito net last night?<br>*Record the names of people who slept under a mosquito net the previous night.*<br>*Question relevant when: ${consent} =1 and ${mosquitonet} =1*	1 All children under 5<br>2 Some of the children under 5<br>3 All household members<br>4 None
usingnet	Since you acquired these mosquito nets, have you treated it/ them?<br>*Here, you are probing if they have applied permethrin to repel mosquitos at night or not*<br>*Question relevant when: ${consent} =1 and ${mosquitonet} =1*	1 Yes<br>0 No
Now we would like to ask you about antenatal care in your most recent pregnacy		
antenatal	In your most recent pregnancy, did you seek antenatal care services?	1 Yes<br>0 No
ancplace	If yes, where did you seek antenatal services from?<br>*Question relevant when: ${consent} =1 and ${antenatal} =1*	1 Professional Health Personnel (Doctor, nurse/midwife, medical assistant/clinical officer)<br>2 Non-professional personnel (traditional birth attendants, community VHT)<br>3 Other (eg, family member)
anctimes	How many times did you go for antenatal care?<br>*Question relevant when: ${consent} =1 and ${antenatal} =1*	1 Four times or more<br>2 Two or Three times<br>3 Once
ancimmune	Did you receive any of the following immunisation and supplements during your antenatal visits?	1 Tetanus injection<br>2 Iron supplements<br>3 IPTP for malaria prevention<br>4 Deworming<br>5 None of the above<br>6 Don't know
malariainpregenancy	During the last pregnancy, did you ever get a fever or malaria?	1 Yes

Field	Question	Answer
		0 No
treatment	If yes, did you seek treatment?<br>*Question relevant when: ${consent} =1 and ${malariainpregenancy} =1*	1 Yes<br>0 No
treatplace	Where did you seek treatment?<br>*Question relevant when: ${consent} =1 and ${malariainpregenancy} =1*	1 Trained health workers/ facility - Government (Government hospital, Government health centre, Outreach services)<br>2 Trained health workers/facility – Private (Private hospital, Private Clinic, Pharmacy)<br>3 Self-medication (Pharmacy/drug shop, shop/market/ domestic herbal medicine)<br>4 Village Health Team/ Community health worker<br>5 Traditional healer/ herbalist
placeofdelivery	Where did you deliver your youngest child from?	1 Kisiizi Hospital<br>2 private clinic<br>3 Government hospital<br>4 Government health centre<br>5 At home<br>6 Along the way to hospital<br>7 Other
deiverymode	How did you deliver?	1 Normal delivery<br>2 Ceaserean section delivery or other
rosterpart3note	Weight of children is an important indicator of how well they are growing. Now, we would like to talk about their weight when they were born. At the end of this section, we shall request to measure their current weight and record it.	
Knowing about the weight of child 1 at birth<br>*Group relevant when: ${consent} =1 and ${userepeat} =1 and ${numfamily} >=1*		
bt_wait_percept1	When [fam_name1] was born, did you think she/he was heavy?	1 Very large<br>2 Larger than average<br>3 Average<br>4 Smaller than average<br>5 Very small

Field	Question	Answer
		6 Don’t know
weighed1	Was [fam_name1] weighed at birth?	1 Yes<br>0 No
baby_weight1	If yes, what was [fam_name1]'s weight at birth?<br>*Read from the birth certificate if its available*<br>*Question relevant when: ${consent} =1 and ${weighed1} =1*	
Knowing about the weight of child 2 at birth<br>*Group relevant when: ${consent} =1 and ${userepeat} =1 and ${numfamily} >=2*		
bt_wait_percept2	When [fam_name2] was born, did you think she/he was heavy?	1 Very large<br>2 Larger than average<br>3 Average<br>4 Smaller than average<br>5 Very small<br>6 Don’t know
weighed2	Was [fam_name2] weighed at birth?	1 Yes<br>0 No
baby_weight2	If yes, what was [fam_name2]'s weight at birth?<br>*Read from the birth certificate if its available*<br>*Question relevant when: ${consent} =1 and ${weighed2} =1*	
Knowing about the weight of child 3 at birth<br>*Group relevant when: ${consent} =1 and ${userepeat} =1 and ${numfamily} >=3*		
bt_wait_percept3	When [fam_name2] was born, did you think she/he was heavy?	1 Very large<br>2 Larger than average<br>3 Average<br>4 Smaller than average<br>5 Very small<br>6 Don’t know
weighed3	Was [fam_name3] weighed at birth?	1 Yes<br>0 No
baby_weight3	If yes, what was [fam_name3]'s weight at birth?<br>*Read from the birth certificate if its available*<br>*Question relevant when: ${consent} =1 and ${weighed3} =1*	
Knowing about the weight of child 4 at birth<br>*Group relevant when: ${consent} =1 and ${userepeat} =1 and ${numfamily} >=4*		

Field	Question	Answer
bt_wait_percept4	When [fam_name4] was born, did you think she/he was heavy?	1 Very large<br>2 Larger than average<br>3 Average<br>4 Smaller than average<br>5 Very small<br>6 Don’t know
weighed4	Was [fam_name4] weighed at birth?	1 Yes<br>0 No
baby_weight4	If yes, what was [fam_name4]'s weight at birth?<br>*Read from the birth certificate if its available*<br>*Question relevant when: ${consent} =1 and ${weighed4} =1*	
postnatalfeeding	Here, we would like to know about post-natal and feeding of the youngest of the children	
We would like to know about postnatal care, especially for the youngest child		
postnatal	Did you do post-natal care, either at home or health facility to monitor your health and the health of the baby?	1 Yes<br>0 No
postnatal_place	If YES, where did you seek post-natal care from?<br>*Question relevant when: ${consent} =1 and ${postnatal} =1*	1 Trained health workers/ facility - Government (Government hospital, Government health centre, Outreach services)<br>2 Trained health workers/facility – Private (Private hospital, Private Clinic, Pharmacy)<br>3 Self-medication (Pharmacy/drug shop, shop/market)<br>4 Village Health Team/ Community health worker<br>5 Traditional healer/ herbalist
breast_feed	For the youngest child (if above 6 months), how long did you exclusively breastfeed?	1 6 months<br>2 More than 6 months<br>3 Less than six months
rosterpart3noteimmunisation	Now, we would like to talk about the immunisation status of the children and other immunity supplements taken	
Immunisation and sickness history of the child<br>*Group relevant when: ${consent} =1 and ${userepeat} =1 and ${numfamily} >=1*		

Field	Question	Answer
immunecard	Could I see [fam_name1]'s immunisation card?	1 Yes, It is available and you can see it<br>2 Yes, I have it but it not available now<br>3 No, I do not have it
childimmune1	Has [fam_name1] been fully immunised with all required immunisation received with in the first 12 months?	1 Yes<br>0 No
childvaccine	Did [fam_name1] receive the following vaccinations - all before he/she was one year of age?	1 BCG- Tuberculosis<br>2 3DPT<br>3 3OPV (Polio)<br>4 Measles<br>5 Other Vaccines
noimmune	Could you tell me why [fam_name1] is not immunised or has not received full immunisation by 1 year?<br>*Question relevant when: ${consent} =1 and ${childimmune1} =0*	1 Lack of information (eg unaware of the need for the 2nd and 3rd doses, fear of side effects)<br>2 Lack of motivation (postponed until another date, no belief in the immunisation, encouraged not to go by friends)<br>3 Obstacles in the system (place for immunisation is too far, the time for immunisation is not convenient, long waiting time, the vaccines are not available, vaccinators nor friendly, costs of immunisation are a lot)<br>4 Obstacles in the family (mother is too busy, poverty - costs of immunisation a lot, family refused)<br>5 Others
VitA	Did [fam_name1] receive Vitamin A supplement in the last 6 months?	1 Yes<br>0 No
Iron_supplement	Has [fam_name1] received any iron supplementation in the last 7 days?	1 Yes<br>0 No
deworm	Was [fam_name1] given any drug for intestinal worms in the last six months?	1 Yes

Field	Question	Answer
		0 No
diarrhoea	Has [fam_name1] had diarrhoea in the last 14 days?	1 Yes<br>0 No
fever	Has [fam_name1] had a fever in the last 14 days?	1 Yes<br>0 No
cough	Has [fam_name1] had a cough in the last 14 days?	1 Yes<br>0 No
treatchild	For all or any of the above illness, did you seek treatment?	1 Yes<br>0 No
treatplace_child	Where did you first seek treatment?	1 Trained health workers/ facility - Government (Government hospital, Government health centre, Outreach services)<br>2 Trained health workers/facility – Private (Private hospital, Private Clinic, Pharmacy)<br>3 Self-medication (Pharmacy/drug shop, shop/market)<br>4 Village Health Team/ Community health worker<br>5 Traditional healer/ herbalist
Now, to finalise the section on health in this household, we would like to ask about knowledge and membership in community health insurance		
costofcare	On your last hospital visit, how much did you pay for hospitalisation and other medical expenses?	
payforhealth	How did you pay for your healthcare for the most recent serious illness/ hospitalisation episode?	1 Paid using household savings<br>2 Sold a household asset to meet healthcare costs<br>3 Borrowed from friends or family member<br>4 Cost was taken care of by family or friends<br>5 Borrowed from a village savings and credit group I participate in<br>6 Paid through community health insurance scheme

Field	Question	Answer
		7 I did not pay, because the services were free of charge at the healthcentre<br>8 Other means
know_abt_cbhi	Have your ever heard of the Kisiizi Hospital Community Health Insurance Scheme?	1 Yes<br>0 No
cbhi_member	Are you currently a member of the KHCHIS?	1 Yes<br>0 No
Yrsin_cbhi	If Yes, For how many years have you been a member?<br>*Question relevant when: ${consent} =1 and ${cbhi_member} =1*	
cbhi_member2010	Were you a member in 2010?	1 Yes<br>0 No
group_name	What is/ was the name of your insurance scheme group in 2010?<br>*Question relevant when: ${consent} =1 and ${cbhi_member2010} =1*	
reason4join	Why did you join the insurance scheme?<br>*Question relevant when: ${consent} =1 and ${cbhi_member} =1*	1 was originally a member of a village tweziike group<br>2 hospital outreach team came to my village<br>3 my parents were members so they recruited me<br>4 was encouraged by friends<br>5 was recruited by neighbours who were forming a group<br>6 I got a health shock which I greatly affected my income and assets<br>7 I can afford the premiums very easily<br>8 I just want to plan for future health eventualities
continue_of_cbhi	Now, as we finalise this section, we would like to get more information on health insurance status	
Now, we would like to know about drop out from cbhi and knowledge of neighbours' status		
notenrolled	Why have you not enrolled in an insurance scheme?	1 I do not have enough information about the scheme<br>2 I do not trust the scheme

Field	Question	Answer
		3 The premiums are expensive<br>4 I did not have enough money for my household needs<br>5 I do not trust the leadership of my group<br>6 The group did not raise the critical number of households<br>7 Conflicts with the leader of a group I wanted to join through<br>8 I do not have any reason to join, (I don't usually fall sick so they would take my money)<br>9 Was discouraged by my colleagues<br>10 Other
drop_out	Have you ever dropped out from the KHCHIS?	1 Yes<br>0 No
dropoutreason	If yes, Why did you drop out of insurance?<br>*Question relevant when: ${consent} =1 and ${drop_out} =1*	1 the scheme was not providing enough information<br>2 The premiums were very high<br>3 I started my own family and dropped from my parents' subscription<br>4 I did not trust the scheme<br>5 Conflicts with the leader of my group<br>6 It is not necessary since we don't always fall sick<br>7 Was discouraged by my colleagues<br>8 Other
four_neighbors	Could you tell me the names (household head) of your four closest neighbours?	
cbhi_neighbor_status	Is any of your neighbours a member of KHCHIS?	1 Yes<br>2 No<br>3 Don't know

Field	Question	Answer
complement_info	Apart from health coverage at the point of care, in which other way have you benefited from being a member of KCHIS?	
We would like to know how you travel to Kisiizi hospital and your nearest other health facility		
modeoftransport	When you travel to Kisiizi hospital, what means of transport do you use?	1 Travel with a car (taxi)<br>2 Travel with a motorcycle taxi (boda boda)<br>3 Travel with a bicycle taxi (boda boda bicycle)<br>4 Walk to the hospital (including stretchers/ engozi)
costoftransport	How much does it cost you to travel to and from Kisiizi hospital?	
time_2_Kisiizihospital	How much time in hours, does it take to travel to and from Kisiizi hospital?	
modeoftransport2	When you travel to your nearest other health centre, what means of transport do you use?	1 Travel with a car (taxi)<br>2 Travel with a motorcycle taxi (boda boda)<br>3 Travel with a bicycle taxi (boda boda bicycle)<br>4 Walk to the hospital (including stretchers/ engozi)
costoftransport2	How much does it cost you to travel to and from your nearest other health centre?	
time_2_otherhealthcentre	How much time in hours, does it take to travel to and from your nearest other health centre?	
waitingtime	In your last hospital visit, how much time in minutes did it take you to see a doctor or nurse to treat you?	
we would like to know about knowledge of TBA ans VHT		
trusted_tba	Do you know any traditional birth attendant (TBA)?	1 Yes<br>0 No
tba_advice	If Yes, Have you sought any medical/ health services and advice from the TBA?	1 Yes<br>0 No
tba_recommend	Would you recommend another person to seek services or advice from the TBA?	1 Yes<br>0 No
vht	Have you received any support or information from the VHT of your village?	1 Yes<br>0 No
We would like to finally talk about how satisfied you were with the care at the hospital		
client_satisfy	On your last visit to Kisiizi hospital as a patient or caretaker, how well do you think you were treated by hospital staff?	1 Was treated with respect

Field	Question	Answer
		2 Felt comfortable with doctors and nurses<br>3 Felt that my privacy was protected<br>4 Doctors and nurses gave me enough time to talk through my condition<br>5 My Expectations were met<br>6 Was treated with negligence<br>7 Hospital staff were disrespectful and inappropriate<br>8 Doctors were on a hurry and did not give me enough time<br>9 My expectations were not met<br>10 Did not have enough trust in the nurses and doctors
satisfy_time	Were you satisfied with the time the doctors and nurses gave you?	1 Yes<br>0 No
satisfy_info	Were you satisfied with the information you received from the doctors and nurses?	1 Yes<br>0 No
satisfy_clinician_conduct	Were you satisfied with the conduct of doctors and nurses?	1 Yes<br>0 No
satisfy_otherstaff_conduct	Were you satisfied with the conduct of other hospital staff?	1 Yes<br>0 No
satisfy_cost	Were you satisfied with the cost you paid for care at the hospital?	1 Yes<br>0 No
satisfy_waitingtime	Do you think the time you spent waiting for a doctor/nurse was appropriate?	1 Yes<br>0 No
sectionnote_household_assets	We have now finished everything concerning health of household members. Now we shall talk about household assets in this household	
hh_assets	Does any member of your household own any of the following assets at present?	1 House<br>2 Other buildings<br>3 Furniture and furnishings<br>4 Refridgerator

Field	Question	Answer
		5 Household appliances (eg, kettle, flat iron etc)<br>6 Television<br>7 Radio<br>8 Solar Panel or electric invertor<br>9 Bicyle<br>10 Motorcycle<br>11 Car<br>12 Mobile phone<br>13 Computer<br>14 Sofa set<br>15 Dining set<br>16 Bed<br>17 Cupboard<br>18 Wall clock
break	We would like to take a short break of about 15 minutes, if you would like to do something quickly such as breastfeeding your child or anything else. When we return, we shall be about half way in our discussion and talk about a few other issues about his household. After the respondent has gone for a break, the interviewer can take a walk around the household, visit the toilets and make observations for the next section	
sectionnote_housing	For some of these questions, please use observation skills and ask only if you are not sure or you have not had the opportunity to observe	
We would now like to ask about housing and other welfare conditions		
external_wall	What is the main type of external wall material used for your house? (Observe)	1 Natural walls (Thatched or straw walls);<br>2 Rudimentary walls (Mud and Poles)<br>3 Rudimentary improved (Unburnt bricks or Burnt bricks with mud)<br>4 Finished Walls (Burnt bricks with cement or Concrete block with cement)<br>5 Other (specify)
roofing	What is the main type of roofing material? (Observe)	1 Grass Thatched<br>2 Corrugated Iron sheets<br>3 Tiles

Field	Question	Answer
		4 Other (specify)
flooring	What is the main type of flooring material? (Observe)	1 Natural Earth or dung<br>2 Cement, stones, wood, marble<br>3 Other
sleeping_rooms	How many rooms does your household use for sleeping?<br>*Response constrained to: .>=0 and .<=5*	
Sectionnote_watsan	Now, we would like to talk about the state of water and sanitation in this household	
We would like to begin with questions about water		
water_source	What is the main source of water for household use (drinking, cooking, washing etc) for members of your household?	1 Protected source (eg from Piped Water, Water from covered well or borehole, Rainwater)<br>0 Unprotected source (eg Water from Open/ unprotected well, Surface Water, River or stream)
why_unprotected_water	What is the main reason for not using protected water sources?	1 Long distance<br>2 Unreliable<br>3 Water does not taste good<br>4 Require contributing money<br>5 Long queues<br>6 Open source is okay<br>7 Other (specify
safe_drinking_water	How do you always make your water safe for drinking?	1 Boil it<br>2 Add water guard or Chlorine<br>3 Strain/seive through a cloth or water filter<br>4 Solar disinfection or Let is stand and settle<br>5 Other<br>6 Do nothing
litres_water	How much water in litres does the household use (for all purposes) per day?<br>*record in litres*	
subsectionnote_sanitation	Here you can use observations taken while you took the 15 minute break and visited the toilets etc. Ask if you are not sure	
Now, we would like to know about toilet facilities		

Field	Question	Answer
bathroom_type	What type of bathroom does this household mainly use?<br>*Observe if posible*	1 Improved bathroom (eg Inside the house, drainage provided; Inside the house, no drainage provided; Outside the house, built and drainage provided)<br>2 Unimproved bathroom (eg Makeshift)<br>3 None (includes outside open space)
toilet_type	What kind of toilet facility do members of your household usually use?<br>*Observe if posible*	1 Improved (eg Flush Toilet; VIP Pit Latrine; Covered pit latrine- with Slab; Covered pit latrine without slab)<br>2 Unimproved (eg Uncovered pit Latrine with slab; Uncovered pit latrine with no slab; Compositing toilet)<br>3 No toilet
hand_wash	Do you have a handwashing facility at the toilet?<br>*Observe if posible*	1 Yes<br>0 No
Sectionnote_energy	Now we would like to move energy use and lighting conditions of this household	
Now we would like to move energy use and lighting conditions of this household		
electricty	Does this household have electricty connection?	1 Yes<br>0 No
lighting	What is the main type of lighting used in the house?	1 Electricity or solar power<br>2 Kerosene Lamp<br>3 Kerosene candle<br>4 Natural Light<br>5 Other
cooking	What is the main type of fuel used for cooking?	1 Electricity or Gas<br>2 Kerosene<br>3 Charcoal<br>4 Firewood<br>5 Dung or Grass<br>6 Other (Specify)

Field	Question	Answer
Sectionnote_labour	Now we would like to know about labour participation for both the husband and wife in this household	
Now we would like to know about labour participation for both the husband and wife in this household		
income_source_hhd	What is your husband’s most important source of earnings during last 12 months?	1 Subsistence farming<br>2 Commercial farming<br>3 Wage employment<br>4 Casual labourer<br>5 Non-agricultural enterprises<br>6 Property income<br>7 Transfers (pension, allowances, social security benefits)<br>8 Remittances (from within the country and abroad)<br>9 Organizational support (e.g. food aid, NGOs etc)<br>10 Other (specify)
full_time_job_hhd	Do you/ Does he usually do this job throughout the year or seasonally?	1 Throughout the year<br>2 Seasonal/part of the year<br>3 Once in a while
income_source_mother	What is your most important source of earnings during last 12 months?	1 Subsistence farming<br>2 Commercial farming<br>3 Wage employment<br>4 Casual labourer<br>5 Non-agricultural enterprises<br>6 Property income<br>7 Transfers (pension, allowances, social security benefits)<br>8 Remittances (from within the country and abroad)<br>9 Organizational support (e.g. food aid, NGOs etc)<br>10 Other (specify)
full_time_job_mother	Do you usually do this job throughout the year or seasonally?	1 Throughout the year<br>2 Seasonal/part of the year<br>3 Once in a while

Field	Question	Answer
Sectionnote_education	Now, we would like to know about education levels of the husband and wife in this household	
Now we would like to know about level of education of the household head and the wife		
educ_level_head	What was the highest level of school you husband finalised?	1 At most completed primary<br>2 At most completed O' Level<br>3 At most completed A' Level<br>4 Tertiary<br>5 University
educ_level_mother	What was your highest level of school you finalised?	1 At most completed primary<br>2 At most completed O' Level<br>3 At most completed A' Level<br>4 Tertiary<br>5 University
why_leave_school	What was your most important reason for your leaving school?	1 Completed desired schooling<br>2 Further schooling not available<br>3 Too expensive<br>4 Too far away<br>5 Had to help at home<br>6 Had to help with farm work<br>7 Had to help with family business<br>8 Poor school quality<br>9 Parents did not want<br>10 Not willing to attend further<br>11 Poor academic progress<br>12 Sickness or calamity in family<br>13 Pregnancy or marriage<br>14 Other
subsectionnote_information	Now, we would like to know about information access in this household	
Now we would like to know about how you access information		
info_source	What is the main source of information for this household?	1 Newspaper<br>2 Radio<br>3 Television<br>4 Mobile phone<br>5 Friends

Field	Question	Answer
		6 Village meetings<br>7 Other
newspaper	How often do you read a newspaper or Magazine?	1 almost every day<br>2 at least once a week<br>3 Less than Once a week<br>4 Never
radio	How often do you listen to radio?	1 almost every day<br>2 at least once a week<br>3 Less than Once a week<br>4 Never
television	Do you watch/ listen to television?	1 almost every day<br>2 at least once a week<br>3 Less than Once a week<br>4 Never
registered_telephone	Does anyone in this household own a mobile phone registered on mobile money?	1 Yes<br>0 No
mobile_money	How often did you use mobile money services in the last 30 days/ one month?	1 At least once every week<br>2 At least once in two weeks<br>3 At least once in a month ago<br>4 Have never used mobile money
Sectionnote_agric	We are almost at the end of our interview with you. We would like to now know about agriculture and related issues in this household	
Here we have the first set of agriculture related questions		
own_land	Does any member of this household own a piece of agricultural land (customary, free hold etc)?	1 Yes<br>0 No
land_size	What is the estimated size of the piece of land?<br>*please record size in acres*	
land_title	Is this land legally titled?	1 Yes<br>2 No,<br>3 Don't Know
fertilizer_use	In the last two cropping seasons, has this household used any fertilisers during cultivation?	1 Yes<br>0 No
pesticides	In the last two cropping seasons, has this household used any pesticides during cultivation?	1 Yes

Field	Question	Answer
		0 No
good_harvest	Would you say you got a very good harvest in your last cropping seasons on your land?	1 Yes<br>0 No
Here we have the second set of agriculture related questions		
farmer_group	Is any member of this household in any farmer's group?	1 Yes<br>0 No
extension_services	Did you or anyone in this household receive advice/information about agricultural/ livestock activities in the past 12 months?	1 Yes<br>2 No<br>3 Don't know
naads_training_participate	Has any member of your household ever participated in a training program organized by NAADS in the past 12 months?	1 Yes<br>0 No
livestock	Does any member of this household own any livestock, (animals or poultry)?	1 Yes<br>0 No
livestock_type	What livestock is owned in this household?	1 Cattle<br>2 Goats<br>3 Sheep<br>4 Pigs<br>5 Chicken<br>6 Other (specify)
Sectionnote_Socialnetworks	This is the last section of this interview with you. We are so grateful for your time this far. The last section will be about the social networks in this household. We therefore want to know about groups you belong to.	
Here, we have the first section of questions on social networks		
groups_preference	Which one of these groups would you prioritise/ prefer to join?	1 Church or religious based groups (eg mothers' union, fathers' union etc)<br>2 Community health insurance group<br>3 Savings and loans association<br>4 Ethnic/clan based group<br>5 Professional association (eg teacher's union)<br>6 Famers' association<br>7 Burial groups

Field	Question	Answer
		8 Other social support groups (eg digging, etc)
membership	Are you a member of any of the above groups or do you know if anyone in this household subscribed to any of the above groups?	1 Yes<br>0 No
group_membership	If Yes, which are the groups in which you or other members of this household subscribe to?<br>*Question relevant when: ${consent} =1 and ${membership} =1*	1 Church or religious based groups (eg mothers' union, fathers' union etc)<br>2 Community health insurance group<br>3 Savings and loans association<br>4 Ethnic/clan based group<br>5 Professional association (eg teacher's union)<br>6 Famers' association<br>7 Other social support groups (eg digging, etc)
group_participation	On a scale of 1 to 5 (where 1 is least and 5 is most), Could you tell me how much you particpate in the groups you belong to?	1 Don’t participate<br>2 Rarely participate<br>3 Averagely participate<br>4 Often participate<br>5 Fully participate
group_barrier1	Have you or anyone in this household ever failed/ refused to join a group you/they desired to join?	1 Yes<br>0 No
group_barrier2	If Yes, which groups did you or anyone in this household fail/ refused to join?<br>*Question relevant when: ${consent} =1 and ${group_barrier1} =1*	1 Church or religious based groups (eg mothers' union, fathers' union etc)<br>2 Community health insurance group<br>3 Savings and loans association<br>4 Ethnic/clan based group<br>5 Professional association (eg teacher's union)<br>6 Famers' association<br>7 Other social support groups (eg digging, etc)

Field	Question	Answer
reason4barrier	Why do you think you were not able to join the group?	1 Did not have the required subscription money<br>2 Did not meet some other non financial requirements<br>3 The group was an exclusive group and I could not join<br>4 Group leadership was not effective<br>5 Conflict between some group members<br>6 Other
here, we have the second section of questions on social networks		
LC_committee	Is anyone in this household a member of LC1, or LC2 or LC3 committee?	1 Yes<br>0 No
public_market_times	How many times in a month do you or a household member goes to your nearest public market?	
comm_participate	In the last one month, have you paticipated in any of these community activities?	1 Wedding, thanksgiving, baptism or other related ceremonies<br>2 Burials ceremonies<br>3 Congregational worship (church or mosque)<br>4 Community planning meetings<br>5 Group digging<br>6 Other
borrow_money	In the last six months, have you borrowed money from an individual or a village savings scheme?	1 Yes<br>0 No
lent_money	In the last six months, have you lent money to individual?	1 Yes<br>0 No
The_End	Thank you very much for this time you have given to us and most importantly, for the information you have provided to us. This information will only be used for this research purposes and will help a lot in understanding how and what impacts CBHI has on the health of mothers and children. We thank you very much.	
