# Supplementary material for: Dropping out of voluntary community-based health insurance in rural Uganda: Evidence from a cross-sectional study in rural south-western Uganda
Source: PLoS One. 2021 Jul 16;16(7):e0253368. doi: 10.1371/journal.pone.0253368 (PMC8284644; doi:10.1371/journal.pone.0253368)
Supplement: S2 File — (DOCX) [file pone.0253368.s002.docx]

| Variable | Variable type | Variable description |
| --- | --- | --- |
| ***Socio-economic controls*** | | |
| Mother’s age | Continuous | Age of the mother recorded in years |
| Father secondary education | Dummy | 1 if father has at least some secondary education and 0 other otherwise |
| Mother secondary education | Dummy | 1 if mother has at least some secondary education and 0 other otherwise |
| Catholic | Dummy | 1 if household is catholic and 0 otherwise |
| Household size | Continuous | Number of individuals residing in the household |
| Father casual employment | Dummy | 1 if father (main income earner) is casually employed and 0 otherwise |
| Household diet diversity score | Continuous | 10 point household diet diversity score based on FANTA scale |
| Food adequacy | Dummy | 1 if respondent thought food was adequate in the last 7 days, 0 otherwise |
| Facility delivery | Dummy | 1 if youngest child was delivered from a health facility and 0 otherwise |
| Attended <=4 ANC visits | Dummy | 1 if mother attended at least 4 antenatal care visits (as recommended) and 0 otherwise |
| Wealth index quintile 1 (Poorest) | Dummy | 1 if household is in poorest quintile and 0 otherwise |
| Wealth index quintile 2 (Poor) | Dummy | 1 if household is in poor quintile and 0 otherwise |
| Wealth index quintile 3 (Average) | Dummy | 1 if household is in average quintile and 0 otherwise |
| Wealth index quintile 4 (Rich) | Dummy | 1 if household is in rich quintile and 0 otherwise |
| Wealth index quintile 5 (Richest) | Dummy | 1 if household is in richest quintile and 0 otherwise |
| ***Social network and perceptions controls*** | | |
| Satisfaction index | Continuous | First principal component from 5 satisfaction questions |
| Burial group size | Continuous | Number of variables in the burial group a household belongs to |
| Neighbour in CBHI | Dummy | 1 if household had a neighbour in CBHI |
| Number of other voluntary groups | Continuous | Number of other voluntary groups that a household belongs to |
| Information access | Dummy | 1 if household has a TV, radio or access to newspapers and 0 otherwise |
| Waiting time | Continuous | Number of minutes in waiting time at the health facility |
| Perception on scheme management | Continuous | First principal component from four questions on management of schemes   1. The management of the scheme has the skill to manage the scheme very well 2. Staff are the scheme are sometimes rude and make people shy from approaching them for help 3. Staff are always helpful and making sure that patients get the best quality care 4. Other hospital staff (such as medical professionals, accounts people and others) treat all patients with equal respect |
| Perception of social influence | Continuous | First principal component from four questions on social influence   1. We learn from out neighbours about the things we do such as which community groups to join 2. Village opinion leaders influence us about the programmes we enrol in, such as insurance 3. Our friends and other extended family members influence our decision to enrol in insurance 4. Enrolling in insurance as an individual household would be better than the current condition to belong in a group 5. The experiences of other community members with insurance affects our decision to enrol in insurance |
| Health beliefs (perceptions) | Continuous | First principal component from four questions on health beliefs   1. Every village (or parish) requires at least one traditional birth attendant 2. Buying health insurance is bad luck, it implies that we are positively embracing/ welcoming sickness in our household 3. Health is a matter in God's hands and insurance cannot help me deal with it. 4. Insecticide treated mosquito nets make sleeping uncomfortable |
| ***Village level controls*** | | |
| No of burial groups in village | Continuous | Number of burial groups in a village |
| Village has a traditional birth attendant | Dummy | 1 if village has a traditional birth attendant, 0 otherwise |
| Village has a health centre | Dummy | 1 if village has a health centre, 0 otherwise |
| Village has a school | Dummy | 1 if village has a school, 0 otherwise |
| Distance to hospital (kms) | Continuous | Distance in kilometres from the village (centre) to the hospital |
| Village altitude (metres) | Continuous | Village altitude in metres above sea level |
