# Supplementary material for: Dropping out of voluntary community-based health insurance in rural Uganda: Evidence from a cross-sectional study in rural south-western Uganda
Source: PLoS One. 2021 Jul 16;16(7):e0253368. doi: 10.1371/journal.pone.0253368 (PMC8284644; doi:10.1371/journal.pone.0253368)
Supplement: S3 File — (DOCX) [file pone.0253368.s003.docx]

Table: S1 - Nested logistic regression model of correlates of dropping out of CBHI with the more restricted assessment of dropping out

|  | (Model 1) | | (Model 2) | | (Model 3) | |
| --- | --- | --- | --- | --- | --- | --- |
| VARIABLES | Odds Ratio | 95% CI | Odds Ratio | Odds Ratio | 95% CI | Odds Ratio |
|  |  |  |  |  |  |  |
| Mother’s age | 0.990 | 0.931 - 1.054 | 1.086** | 1.006 - 1.173 | 1.106* | 0.999 - 1.226 |
| Father secondary education | 5.983*** | 1.664 - 21.512 | 10.216*** | 2.204 - 47.352 | 16.760** | 1.258 - 223.227 |
| Mother secondary education | 1.945 | 0.505 - 7.485 | 1.746 | 0.331 - 9.199 | 0.990 | 0.177 - 5.534 |
| Catholic | 0.695 | 0.271 - 1.787 | 0.931 | 0.324 - 2.676 | 1.263 | 0.403 - 3.954 |
| Married | 0.414 | 0.093 - 1.851 | 0.201 | 0.027 - 1.513 | 0.201 | 0.028 - 1.448 |
| Household size | 1.454*** | 1.115 - 1.895 | 1.173 | 0.866 - 1.587 | 1.195 | 0.857 - 1.665 |
| Father casual employment | 0.763 | 0.266 - 2.187 | 0.870 | 0.261 - 2.895 | 0.953 | 0.181 - 5.006 |
| HDDS | 1.055 | 0.755 - 1.474 | 1.051 | 0.755 - 1.462 | 0.798 | 0.466 - 1.367 |
| Food adequacy | 0.601 | 0.212 - 1.702 | 0.600 | 0.200 - 1.805 | 0.342* | 0.108 - 1.077 |
| Wealth index (base Q5 -richest) |  |  |  |  |  |  |
| Quintile 1 (poorest) | 5.564** | 1.287 - 24.060 | 4.502* | 0.764 - 26.524 | 9.922* | 0.933 - 105.518 |
| Quintile 2 | 5.546** | 1.217 - 25.280 | 7.683** | 1.313 - 44.949 | 23.599*** | 2.145 - 259.623 |
| Quintile 3 | 0.252 | 0.023 - 2.730 | 0.251 | 0.007 - 9.597 | 0.955 | 0.010 - 95.061 |
| Quintile 4 | 0.655 | 0.151 - 2.837 | 0.835 | 0.114 - 6.126 | 2.078 | 0.194 - 22.294 |
| Information access | 0.576 | 0.218 - 1.521 | 0.536 | 0.171 - 1.678 | 0.625 | 0.207 - 1.885 |
| Waiting time | 1.000 | 0.996 - 1.003 | 1.002 | 0.998 - 1.007 | 1.002 | 0.996 - 1.008 |
| Satisfaction index |  |  | 0.920 | 0.592 - 1.430 | 0.976 | 0.530 - 1.800 |
| Burial group size |  |  | 1.022** | 1.003 - 1.041 | 1.059*** | 1.027 - 1.093 |
| Neighbour in CBHI |  |  | 0.271** | 0.088 - 0.831 | 1.401 | 0.272 - 7.219 |
| Number of other voluntary groups |  |  | 0.151*** | 0.059 - 0.386 | 0.144*** | 0.049 - 0.421 |
| Perceptions about CBHI |  |  |  |  |  |  |
| Scheme management |  |  | 1.104 | 0.759 - 1.604 | 1.181 | 0.682 - 2.046 |
| Social influence |  |  | 0.993 | 0.720 - 1.370 | 0.853 | 0.587 - 1.239 |
| Health beliefs |  |  | 0.959 | 0.511 - 1.801 | 0.703 | 0.290 - 1.704 |
| No of burial groups in village |  |  |  |  | 0.566*** | 0.385 - 0.832 |
| Village has a traditional birth attendant |  |  |  |  | 2.320 | 0.549 - 9.796 |
| Village has a health centre |  |  |  |  | 0.291 | 0.037 - 2.293 |
| Village has a school |  |  |  |  | 19.869*** | 3.145 - 125.537 |
| Distance to hospital (kms) |  |  |  |  | 1.511** | 1.059 - 2.157 |
| Village altitude (metres) |  |  |  |  | 1.000 | 0.992 - 1.009 |
| Constant | 0.030*** | 0.004 - 0.224 | 12.960* | 0.683 - 245.920 | 0.473 | 0.005 - 42.135 |
|  |  |  |  |  |  |  |
| Observations | 233 |  | 233 |  | 233 |  |
| Mean VIF | 3.28 |  | 4.18 |  | 4.32 |  |
| R-squared | 0.203 |  | 0.401 |  | 0.544 |  |

Robust standard errors in parentheses

*** p<0.01, ** p<0.05, * p<0.1
