# Supplementary material for: Dropping out of voluntary community-based health insurance in rural Uganda: Evidence from a cross-sectional study in rural south-western Uganda
Source: PLoS One. 2021 Jul 16;16(7):e0253368. doi: 10.1371/journal.pone.0253368 (PMC8284644; doi:10.1371/journal.pone.0253368)
Supplement: Attachment — Submitted filename: Rebuttal letter - Response to editor & Reviewer.docx [file pone.0253368.s004.docx]

Dear Prof Khan,

We appreciate the feedback on our manuscript *PONE-D-20-31908R1 - [EMID:0b93724483f837a2]* which has gone through the review process at PLOS ONE. We have received very helpful comments from two reviewers in the first round review and another insightful comment from the second round review. We sufficiently responded to first round review comments and now provide a response to the second round comment. We are very pleased that all the reviewers through the rounds found our manuscript promising and of academic and policy interest. Unfortunately, we believe that decision to reject might have emanated from one key area of the manuscript that was not well elaborated. We appreciate that the reviewer gave us a chance of resubmitting a revised manuscript and we are very glad to resubmit the manuscript. In the following rebuttal, we provide a point by point response to each concern raised by the reviewer. Our responses are provides in italics to be easily distinguished from the reviewer’s comments.

**Response to comments**

The paper has promise, especially the quantitative part of the paper. The inclusion of qualitative aspect appears to be an after-thought and is not well-integrated with the overall flow of the paper. Probably because of the inclusion of qualitative results, the authors did not provide adequate explanations of the quantitative models… The authors may decide to submit a new paper using the quantitative results only and by modifying the dependent variable. The empirical results and implications of the parameter estimates need careful rethinking and explanation.

*We thank the reviewer for this comment. In light of this comment, we have revised the manuscript, omitting the qualitative results and focusing the manuscript only on the quantitative aspect. Moreover, we have also changed the title of the manuscript. The current manuscript is: “****Dropping out of voluntary community-based health insurance in rural Uganda: Evidence from a cross-sectional study in rural south-western Uganda.”*** *We believe this is a more appropriate title.*

Readers would like to know more about the comparisons of the three models in the paper and how to interpret the results for understanding the factors associated with dropping out of insurance.

*We have revised the data analysis section to be clear on how our models are constructed and how they should be interpreted. In a summary, we conduct logistic regression models in a hierarchical manner.*

One result seems to be consistent across the three models is that the wealth index affect the probability of dropping out of insurance. It is probably not surprising that those who do not drop out are more likely to use health facilities, receive 4+ antenatal care or delivery in health facilities. One can interpret these relationships not as factors affecting continued enrollment but the propensity of households to remain in the insurance program if they intend to use services from health facilities. If this is true, there should be quite significant proportion of households who would be participating and dropping out and then coming back to get insurance based on projected health care needs. Ever dropped from insurance as the dependent variable will not be the right variable to use in this situation.

*This is a very profound comment which we address in two ways.*

*First, we omit two control variables (4+ ANC visits and delivery at a health facility) from the model. The omission of these variables does not affect any of the other variables in either direction or magnitude. We therefore agree with the reviewer that their omission is correct.*

*However, the reviewer also alludes to moral hazard when they say that individuals might enrol with the intent of using services and drop out when they no longer need immediate services, especially in the face of high enrolment fees (premiums). I a previous study, we elaborately discuss the issue of moral hazard* (Nshakira-Rukundo, Mussa, Nshakira, Gerber, & von Braun, 2021)*. This particular scheme also sufficiently deals with moral hazard by having an extremely long waiting time before full coverage. To put this in context, the scheme institutes three levels of waiting before full coverage. New enrolees (including those having gaps in enrolment) wait for 30 days before they receive any kind of coverage. This is at par with the CBHI scheme in Nigeria (Bonfrer, Poel, Gustafsson-Wright, & Doorslaer, 2018). Between the second and 11th month after initial enrolment, the scheme covers only 10% of health costs. The scheme covers all insured costs only after the 12th month/ 1 year after full enrolment.*

*In addition, we would like to clarify on how the dependent variable “dropping out of CBHI” was measured. In the survey, conducted in 2015, we asked respondents if they had been enrolled in 2015 and if they had been enrolled in 2010. The difference in enrolment status between these two time periods provides one dimension of dropping out. This is the more restrictive measure. In a less restrictive measure, we also asked individuals if they ever dropped out pre-2010. We therefore believe that a less restrictive measure is preferred for both its slightly larger sample size (n=251 compared to n=232) and that we are able to observe a slightly longer time trend of enrolment behaviour. However, we also provide results of the more restrictive in supplementary results. The results are not so much distinguishable in inference however, due to a smaller sample, the standard errors are larger and the confidence intervals are wider and therefore this model is less preferred.*

The authors may decide to submit a new paper using the quantitative results only and by modifying the dependent variable.

*We highly welcome the reviewer’s recommendation of a revised paper and are very glad to resubmit this manuscript.*

*We look forward to your kind response.*

With kind regards,

Dr Cho, Minjung,

Dr Mussa, Essa Chanie,

Dr Nshakira-Rukundo, Emmanuel

**Cited works**

Bonfrer, I., Poel, E. Van De, Gustafsson-Wright, E., & Doorslaer, E. Van. (2018). Voluntary health insurance in Nigeria : Effects on takers and non-takers. *Social Science & Medicine*, *205*, 55–63.

Nshakira-Rukundo, E., Mussa, E. C., Nshakira, N., Gerber, N., & von Braun, J. (2021). Impact of community-based health insurance on utilisation of preventive health services in rural Uganda: a propensity score matching approach. *International Journal of Health Economics and Management*. https://doi.org/10.1007/s10754-021-09294-6
